# Oral cholera vaccine use in Zanzibar: socioeconomic and behavioural features affecting demand and acceptance

**DOI:** 10.1186/1471-2458-9-99

**Published:** 2009-04-07

**Authors:** Christian Schaetti, Raymond Hutubessy, Said M Ali, Al Pach, Mitchell G Weiss, Claire-Lise Chaignat, Ahmed M Khatib

**Affiliations:** 1 Department of Public Health and Epidemiology, Swiss Tropical Institute, PO Box, Socinstrasse 57, 4002 Basel, Switzerland; 2 Initiative for Vaccine Research, World Health Organization, 20, avenue Appia, 1211 Geneva 27, Switzerland; 3 Public Health Laboratory Ivo de Carneri (PHL-IdC), Ministry of Health and Social Welfare of Zanzibar, PO Box 122, Chake-Chake, Pemba, United Republic of Tanzania; 4 International Vaccine Institute, SNU Research Park, San 4-8, Bongcheon-7-dong, Kwanak-gu, Seoul, 151-919, Korea; 5 Global Task Force on Cholera Control, World Health Organization, 20, avenue Appia, 1211 Geneva 27, Switzerland; 6 Ministry of Health and Social Welfare of Zanzibar, PO Box 236, Zanzibar, United Republic of Tanzania

## Abstract

**Background:**

Cholera remains a serious public health problem in low-income countries despite efforts in the past to promote oral rehydration therapy as major treatment. In 2007, the majority of worldwide cases (94%) and deaths (99%) were reported from Africa. To improve cholera control efforts in addition to maintaining and improving existing water supply, sanitation and hygiene behaviour measures, the World Health Organization has recently started to consider the use of vaccines as an additional public health tool. To assess this new approach in endemic settings, a project was launched in Zanzibar to vaccinate 50,000 individuals living in communities at high risk of cholera with an oral two-dose vaccine (Dukoral^®^).

Immunisation programmes in low-income countries have suffered a reduced coverage or were even brought to a halt because of an ignorance of local realities. To ensure the success of vaccination campaigns, implementers have to consider community-held perceptions and behaviours regarding the infectious disease and the vaccine of interest.

The main aim of this study is to provide advice to the Ministry of Health and Social Welfare of Zanzibar regarding routine introduction of an oral cholera vaccine from a socioeconomic and behavioural perspective as part of a long-term development for a sustained cholera prevention strategy.

**Methods and design:**

Qualitative and quantitative methods of health social science research will be applied on four stakeholder levels before and after the mass vaccination campaign. Rapid assessment individual interviews and focus groups will be used to describe cholera- and vaccine-related views of policy makers, health care professionals and community representatives. The cultural epidemiological approach will be employed on the individual household resident level in a repeated cross-sectional design to estimate determinants of anticipated and actual oral cholera vaccine acceptance.

**Discussion:**

The study presented here is designed to inform about people's perceptions regarding cholera and about socioeconomic and behavioural factors determining anticipated and actual oral cholera vaccine acceptance in Zanzibar. Its pre- and post-intervention design using a mixed-methods approach on different stakeholder levels in communities at high risk of cholera outbreaks will ensure the collection of locally valid data relevant for public health action and planning.

## Background

### Etiology, symptoms and treatment of cholera

Cholera is an intestinal disease caused by the bacterium *Vibrio cholerae *which spreads mainly through faecal contamination of water and food by infected individuals [1]. Eating raw or undercooked seafood can also cause the infection since *V. cholerae *was found on phyto- and zooplankton in marine, estuarine and riverine environments independent of infected human beings [2,3]. Two out of ca. 240 serogroups of *V. cholerae *– O1 and O139 – are pathogenic. The O1 serogroup can further be subdivided into two biotypes – classical and El Tor.

After an incubation period of 18 hours to five days, infected individuals will develop acute watery diarrhoea. Large volumes of rice-water-like stool and concurrent loss of electrolytes can lead to severe dehydration and eventually death if patients are not rapidly treated. Most of the infected individuals, however, are asymptomatic or suffer only from mild diarrhoea. An inoculum of 10^8 ^bacteria is needed in healthy individuals to cause severe acute watery diarrhoea while a 1,000-fold lower dose is sufficient to cause the disease when gastric acid production is reduced. Other clinical features besides profuse diarrhoea (more than three loose stools per day) to establish a cholera diagnosis include abdominal and muscle cramps and frequent vomiting [4]. Without treatment the case-fatality rate (CFR) can reach 50% [1].

Treatment of cases depends on the severity and includes i) giving oral rehydration solutions (ORS) after each stool if no dehydration is apparent, ii) giving ORS in larger amounts if moderate dehydration is apparent, and iii) using intravenous drips of Ringer Lactate or saline for severely dehydrated patients [4]. Antibiotics can be administered to shorten the episode in severe cases.

### Prevention of cholera

Cholera usually occurs in epidemics and can cause major disruptions in affected health systems as rigorous measures have to be taken and patients treated in camps under quarantine-like conditions. Outbreaks of cholera can easily be prevented by providing safe water, sanitation and promoting good personal hygiene behaviour and safe food handling. Regions where such control measures have not been realised, or where maintenance and monitoring of existing schemes is not guaranteed, are at greatest risk of epidemics and consequently could become endemic with cholera.

The World Health Organization (WHO) has recently started to consider the use of vaccines as an additional public health tool to control cholera in low-income countries since the implementation of the above-mentioned prevention and control measures has not had the desired impact on cholera incidence [5]. Currently only one safe and efficacious vaccine is available on the market – Dukoral^® ^– an oral cholera vaccine (OCV) consisting of killed whole-cell *V. cholerae *O1 with purified recombinant B-subunit of cholera toxoid. It has to be administered in two doses about one week apart. It confers, as shown in field trials in Bangladesh, Peru and Mozambique, 60–85% protection for six months in young children and about 60% in older children and adults after two years [6-8]. Longini Jr. et al. [9] used data collected in 1985–1989 from a randomised controlled OCV trial in Bangladesh to calculate reductions of cholera cases. Their model indicated that a 50% coverage with OCV would lead to a 93% reduction in the entire population while a lower coverage of 30% would still reduce the cholera incidence by 76%.

### Global and local burden of cholera

Cholera is mainly endemic in low-income countries in Africa, Asia, Central and South America. A total of 177,963 cases and 4,031 deaths, corresponding to a CFR of 2.3%, have been reported to WHO in 2007 with Africa having the largest share of worldwide reported cholera cases (94%) and deaths (99%) [10]. This share of officially reported cases from Africa has increased considerably from 20% in the 1970s to 94% in the period 2000–2005 while the Asian share has simultaneously dropped from 80% to 5.2% over the same three decades [11]. There is a similar picture with regard to reported deaths: Africa's share has increased from 22% to 97%, and Asia's has showed a steep decline from 77% to 2.4%. It has to be noted, however, that these official figures do not reflect the true burden of cholera since serious under-reporting due to technical (surveillance system limitations, problems with case definition and lack of standard vocabulary) and political (fear of travel or trade sanctions) reasons are suspected [10]. Zuckerman et al. [12] identified mainly under-reporting from the Indian subcontinent and Southeast Asia in a review carried out in 2004.

In Zanzibar, where this study will be conducted, a cholera outbreak with 411 cases and 51 deaths was reported for the first time in 1978 from a fishermen village [13]. Thirteen outbreaks followed since then with almost annual episodes since the year 2000, with case-fatality rates ranging from 0% to 17% and showing a downward trend over the last two decades (Reyburn et al., unpublished data). During the last outbreaks in 2006/2007, 3,234 cases and 62 deaths were reported (CFR: 1.9%). A seasonal pattern can be observed that follows the rainy seasons (usually from March to June and from October to November) during which widespread flooding occurs frequently. Such deteriorating environmental conditions subsequently expose the majority of inhabitants on both islands to an increased risk of water-borne diseases due to the scarcity of safe drinking water supplies and a generally poor or lacking sanitation infrastructure in periurban and rural areas.

Despite all efforts in the past and the inexpensive and relatively easy use of ORS as major treatment [14], cholera still poses a serious public health problem in low-income countries. Thus, a concerted action is needed to control cholera and to mitigate its health-related and economic consequences not only by maintaining and improving existing measures like water supply, sanitation and hygiene behaviour but also by assessing new prevention options like OCV mass vaccinations of vulnerable populations [5,12,15].

### Importance of sociocultural and behavioural research on vaccine introduction

Public health interventions to reduce disease burden must take into account the local realities to achieve a sustainable benefit for the affected populations. Solely relying on prevention or treatment measures that proved to be suitable in a given context does not necessarily make it appropriate for other situations.

Vaccination programmes have suffered a reduced coverage (e.g. rumours about tetanus toxoid causing infertility in Tanzania [16]) or were even brought to a halt because of an ignorance of local realities (e.g. Northern Nigerian resistance to polio vaccination [17,18]). Other interventions, especially when implemented in a top-down approach, experienced the same difficulties [19-21].

To ensure the success of vaccination campaigns, a vaccine should not only be efficacious, relatively trouble-free for patients in its administration and preferably also cost-effective, but it is equally important that implementers consider community-held ideas, fears and individual help-seeking behaviour regarding the infectious disease and the vaccine of interest [22,23].

Infrastructure, logistics, politics, and social and cultural features were identified as significant factors which determine vaccine acceptance and thus the success or failure of immunisation programmes in low-income countries [24-26]. The importance of the social and cultural context on vaccine acceptance was assessed in various recent studies for typhoid fever and shigellosis (e.g. in Asian countries [27-31]). It was reasserted that the importance of the social and cultural context on vaccine introduction has to be studied carefully in order to improve vaccination coverage [32].

Research to improve the health of people needs to include gender issues since they play a crucial role in health and health planning [33]. Gender differences are context-specific and thus require that sociocultural and behavioural research be done to complement clinical or epidemiological research. A recent review on the control of tropical diseases concluded that more detailed data about illness experience, meaning and help-seeking behaviour is needed on the gender level to inform the planning and execution of health interventions [34].

Socioeconomic features, and cultural beliefs and practices, may vary across and within different sites or populations. And since differences in income, education, neighbourhood, infrastructure, etc. can affect people's health and behaviour regarding risk and relief, it is prudent to include site-specific analyses when doing research on the acceptance of community vaccine interventions.

### Protocol review and ethical clearance

This paper summarises the protocol that had been reviewed by two independent scientists with expertise in the field of cholera and social science research before it was submitted to and accepted by the WHO Research Ethics Review Committee and the Ethics Committee of Zanzibar.

All participants will be informed about the study and individual written consent obtained before conducting discussions or interviews. All data will be handled with strict confidentiality and made anonymous before analysis.

### Rationale for research: socioeconomic and behavioural (SEB) study

In late 2006, WHO received a grant from the Bill and Melinda Gates Foundation to work on the pre-emptive use of OCV in vulnerable populations at risk. The main focus of this grant is to examine how OCV can sustainably be used in countries with endemic cholera in addition to usually recommended control measures such as provision of safe water, adequate sanitation and health education. An important feature of the project is to collect evidence to assess the usefulness and financial stability of establishing an OCV stockpile.

**Figure 1:**
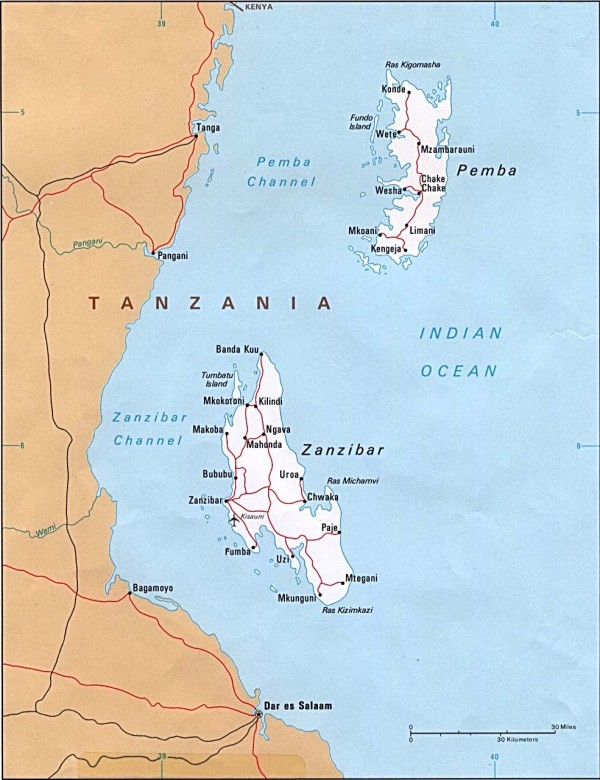
**Map of Zanzibar with the two main islands**. Courtesy of the University of Texas Libraries. The University of Texas at Austin.

To achieve these goals, WHO launched a joint venture with the Ministry of Health and Social Welfare of Zanzibar (MoHSW) to vaccinate 50,000 community residents older than two years living in communities at high risk of cholera with Dukoral^®^. The two islands of Zanzibar (Figure 1) were chosen as study area since they have been regularly affected by cholera over the past three decades and since the local government wishes to enhance its strategy to control the disease and to examine the possibility of introducing OCV as a public health measure.

Complementary to the vaccination campaign, since no sociocultural and behavioural studies related to cholera and OCV introduction have been conducted yet in African settings, the SEB study was conceived as a pilot project to address the research questions stated below. Besides the focus on cholera, it was also decided to include, to a lesser extent, shigellosis (bloody dysentery caused by *Shigella spp*.) in this research to investigate similarities and differences between the community perceptions of these two serious and potentially fatal diarrhoeal diseases.

### Aims and research questions

The main aim of the SEB study, its stakeholders and research questions are as follows:

To generate evidence on the role socioeconomic and sociocultural factors can play to inform government policies regarding the introduction of OCV as part of a sustainable and financially viable cholera control strategy.

To inform the Government of Zanzibar, in particular the Ministry of Health and Social Welfare, regarding the national policy on cholera control and the use of OCV on the archipelago.

#### Stakeholders

Research will be done on the following four stakeholder levels in Zanzibar:

- Level I: policy makers on national and regional level

- Level II: allopathic and traditional health care providers working in the target areas and district hospitals

- Level III: formal and informal local government and community leaders and teachers from the target areas

- Level IV: adult community residents (household level)

#### Research questions

In populations where cholera is endemic:

- What are the perceptions of cholera in the context of diarrhoeal diseases, in particular shigellosis?

- What are the essential features of cholera and shigellosis?

- What is the acceptance of OCV?

For each question, the following comparisons will be made between:

- Gender

- Site: periurban (Unguja) vs. rural (Pemba)

- Vaccination (intervention) status

- Stakeholders: all four levels

## Methods and design

### Study setting

Zanzibar consists of two major islands, Unguja (also named Zanzibar) and Pemba. They are situated in the Indian Ocean about 40–60 km off the coast of Tanzania and a few degrees south of the equator (Figure 1). In 1964, shortly after independence from the colonial powers, the archipelago and Tanganyika formed the United Republic of Tanzania. Zanzibar as a semiautonomous entity within Tanzania consists of five regions which are subdivided into ten districts, 50 constituencies and 296 communities (Shehias). The main islands cover approximately 2,557 km^2 ^(Unguja: 1,651 km^2^, Pemba 906 km^2^). They are inhabited by a population of approximately 985,000 (in 2002) with Unguja having a share of 63% and Pemba of 37%. The inter-censal annual growth rates (1988–2002) varied from 2.1 to 4.5% [35]. Its mainly Islamic inhabitants are speaking Kiswahili. One-fourth of the population has not received any education and the primary education net enrolment ratio amounts to 77% [36].

The majority of the population (71%) is having access to piped water while a minority has to rely on drinking water from wells (27%) and other sources like street vendors, rainwater, spring water, and open water courses (2%) [36]. About half of the population (53%) has access to pit latrines, while more than one-fourth has no toilet facility (28%) and 12% – mainly in urban areas – are using a flush toilet.

The top three causes of admission to Zanzibar hospitals in 2007 were malaria (27.4% of all admissions), gastroenteritis (12.7%) and pneumonia (9.9%) [37]. The main causes of death were malaria (18.4%), hypertension (8.5%) and pneumonia (7.9%) with gastroenteritis ranking on fourth place (7.5%). The main sources of help consulted are primary health care units which are situated within four kilometres of households for over 90% of the population. Monthly mean per capita expenditure was TZS21,000 (ca. USD18) in 2004/5 with a 2.1% share for health-related expenditures [36]. Life expectancy at birth has risen from 47 years in 1988 to 57 years in 2002 [35].

#### Target areas

The SEB study will take place in the periurban Shehia of Chumbuni (population estimate: ca. 13,500) in Unguja and the rural Shehia of Mwambe (population estimate: ca. 8,500) in Pemba. These two Shehias represent core areas of the mass vaccination campaign and were selected based on epidemiological data collected from recent cholera outbreaks (Reyburn et al., unpublished data) [13]. The selection of Chumbuni and Mwambe as target areas for the SEB study was therefore based on epidemiological vulnerability criteria (i.e. attack rates) and not on socioeconomic status.

### Methodological framework

The research questions will be answered by using both quantitative and qualitative methods suited for the different stakeholder levels. On levels I to III rapid assessment methods will be used [38,39] while on level IV the cultural epidemiological approach using Explanatory Model Interview Catalogue (EMIC) interviews will be employed [40]. Focus group discussions (FGD) will also be conducted as preparation for EMIC interviews.

#### Level I to III: rapid assessments

Rapid assessments have been utilised to address various health issues including malaria, sexually transmitted diseases, diarrhoeal disease, water and sanitation, and nutrition to name a few. Rapid assessments are methodologically tied to the basic tenets of anthropological ethnographic research which emphasises the use of multiple sources of data to gain various perspectives on social phenomena.

The rapid assessments in this study will provide the meanings and experiences of how community leaders, health care providers and policy makers perceive the importance of cholera as a public health concern and the need and demand for a cholera vaccine. These data will be important for future public health implementation of an oral cholera vaccination programme.

#### Level IV: cultural epidemiology

Cultural epidemiology incorporates qualitative and quantitative methods of health research and uses culturally adapted EMIC interviews to elicit locally valid representations of illness-related experience, meaning and behaviour [41]. Like classical epidemiology, cultural epidemiological research can focus on descriptive, analytical or comparative questions with regard to control of a disease or other public health interests. Analytical studies in cultural epidemiology consider the impact local categories of distress, perceived causes and help seeking – similar to the epidemiological risk factors – are having on clinical or public health outcomes.

A first concept of this research approach was already proposed 20 years ago to the benefit of health planning within the context of diarrhoeal illnesses and ORS promotion [42]. An early validation of this approach was later done in central Thailand when EMIC interviews were used to describe local diarrhoeal illnesses for generating public health policy recommendations [43]. In this study, EMIC interviews will be used to describe the cultural epidemiology of cholera and shigellosis and to estimate determinants of OCV acceptance.

**Figure 2:**
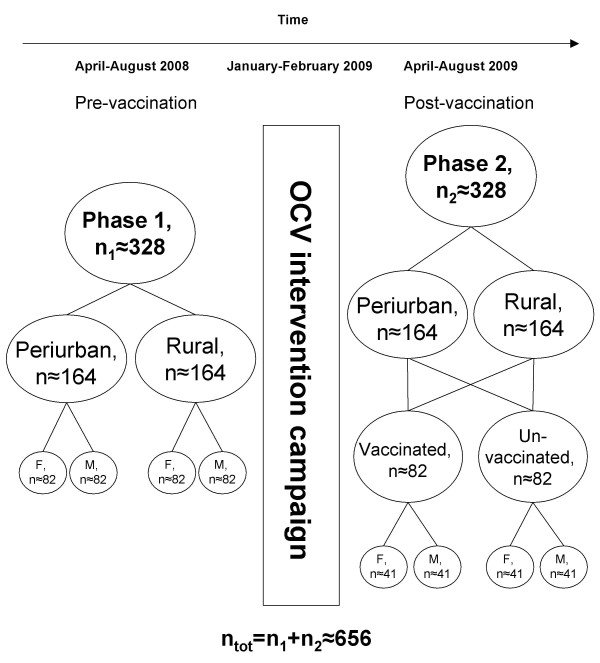
**Outline of the repeated cross-sectional design for household-level study**. Not shown is the preparatory phase taking place before phase 1. F: Female, M: Male.

A repeated cross-sectional cultural epidemiological study will be conducted in the two target areas (Figure 2). After a preparatory phase, where focus group discussions will be held, a baseline survey using EMIC interviews (phase 1) will be done in a random sample from each Shehia. After the intervention new samples will be drawn randomly based on the vaccination status. The same instrument will then be used again but with minor changes to account for the post-intervention status of the communities (phase 2).

##### Focus group discussions

Focus group discussions will be used to describe the context of cholera and shigellosis in the target areas. Results from the FGD among community residents will help to guide the planned quantitative research, i.e. the FGD guideline follows the topics and items that will be elicited in the EMIC interviews described below.

##### EMIC interviews

The interview will enquire about the sociocultural context in terms of illness-related experience, meaning and help-seeking behaviour and about topics related to vaccination. Under experience, different aspects of the illness like physical symptoms and psycho-emotional and social problems and also financial issues that can have an impact on patients will be elicited. Under meaning, respondents will be questioned about their views and opinions on why and how they think one can get the illness with regard to various categories of perceived causes including biological, behaviour-related, social and traditional/magico-religious factors. To find out more about sources of help seeking, respondents will be asked to identify and assess all health care providers, i.e. locally available allopathic and traditional sources, patients suffering from cholera or shigellosis will likely consult. The possible options for self-treatment (at home, not at a health-care provider) will also be elicited.

Variables to be elicited will include:

- Sociodemographic factors

- Categories of diarrhoeal illnesses, name of condition

- Perceived severity of cholera and shigellosis episodes

- Perceived vulnerability of cholera and shigellosis

- Illness experience: operationalised as patterns of distress (PD) related to cholera and shigellosis

- Illness meaning: operationalised as perceived causes (PC) related to cholera and shigellosis

- Illness behaviour: operationalised as help seeking (HS) within and outside the household related to cholera and shigellosis

- Stigma (index) of cholera

- Prior episodes of cholera

- Prevention of cholera

- Experience and perception of vaccinations

- Barriers to vaccination (only phase 2)

- Anticipated vaccination status (only phase 1)

- Willingness-to-pay for OCV

- Actual vaccination status, i.e. vaccinated/unvaccinated, not to be elicited but recorded during vaccination campaign

##### Sample size calculation for EMIC interviews

The following calculations are based on a 95% significance level and 80% power. The prominence means of the different categories of cultural epidemiological variables will be compared in bivariate analyses (by site, gender, anticipated vs. actual vaccination status) and tested for significant differences using the Wilcoxon rank-sum test. To detect a difference of 0.5 between prominence means with equal standard deviations of 1.5, a sample size of 164 for each group is required. The calculation is based on a two-sample t-test assuming a worst-case scenario, i.e. no underlying distribution in the data, which requires that the sample size derived from the t-test (n = 142) be divided by 0.864 which equals 164.4 [44]. Hence, the overall sample size (two groups per phase times two phases) will be 656, with a sample size of 328 per phase.

### Data collection

#### Level I to III

##### Strategy

Rapid assessment individual interviews and focus groups will collect qualitative information on policy makers' considerations and need for information for planning the introduction of a cholera vaccination programme. This phase of the study will also involve data collection with health care providers and community leaders on their perceptions of cholera, its importance to community residents, and their interest in the OCV. This research will explore policy issues and processes, as well as barriers and enabling factors that are likely influences in the introduction of a vaccine against cholera. The descriptive data of the rapid assessment interviews and focus groups can be related to and compared with level IV data. The approximate duration of this phase of the study is four weeks. It will be conducted following the mass OCV campaign in order to ground perspectives on the acceptance of an OCV in real world contexts and experiences.

##### Instrument

In-depth interviews and group discussions are open-ended and semi-structured to both cover critical topics and allow the respondents to identify and discuss what is important to them regarding cholera and the use of a cholera vaccine. For each stakeholder level specific interview and discussion guidelines have been created, translated and orally back-translated to ensure their intelligibility and appropriateness. Two experienced field researchers will conduct the research either in English or Kiswahili depending on the respondents. Each session will last approximately one hour.

##### Sampling

The following groups will be selected purposively:

- Policy makers (n = 10–20) including national and regional policy makers (e.g. from MoHSW, international non-governmental organisations);

- Allopathic and traditional health care providers (n = 6–8 per island) working in the target areas and district hospitals;

- Formal and informal local government and community leaders and teachers (n = 6–8 per island) from the target areas (e.g. Shehas, secular and Islamic teachers).

#### Level IV: preparatory phase

##### Strategy

Community residents will be approached on both islands and information collected in focus group discussions. Data will be collected to complete or expand the lists of variables of the different EMIC interview sections mentioned above. The approximate duration of this phase is two weeks with one team working per site. This phase must be completed before phase 1 starts.

##### Instrument

A FGD guideline was drafted which covers all the relevant issues of the EMIC interviews. Each team will consist of a moderator and a note taker.

##### Sampling

- Sample: adults (≥ 18 years) from both communities;

- Sampling frame: registers from Shehias adjacent to the target Shehias;

- Sampling method: stratified purposive sample, by gender and age group (18–45 years, >45 years);

- Exclusion criteria: belonging to other stakeholder level I to III;

- Sample size: n = 48–64, eight FGD with each having ca. 6–8 respondents (4 FGD per site)

#### Level IV: phase 1 (pre-vaccination)

##### Strategy

Community residents will be approached on both islands and information collected through EMIC interviews. Data will be collected i) to clarify the local features of cholera and shigellosis; and ii) to identify social and cultural determinants of anticipated OCV acceptance. The approximate duration of this phase is about ten weeks. Three teams each per site will be working simultaneously. This phase must be completed before the social mobilisation and vaccination campaign starts.

##### Instrument

An EMIC interview for phase 1 was drafted and, once it has incorporated information from the preparatory phase (FGD), will be pilot tested and finalised before implementation. Since the general adult population – and not cases – will be interviewed, it was decided to introduce the topic to the interviewees by using slightly varying gender-specific vignettes which describe cardinal physical symptoms of cholera and shigellosis. This approach ensures that the interviewees will respond with regard to the clinically relevant conditions and not with regard to local notions of diarrhoeal illnesses labelled by the respective local terms for cholera and shigellosis.

A ten-day workshop will be conducted to train the fieldworkers in interview and data entry skills, to familiarise them with the instrument and to pilot it under field conditions. Each team will consist of an interviewer and a note taker. Narrative data will be translated and typed on a daily basis by the teams.

##### Sampling

- Sample: adults (≥ 18 years) from both target areas;

- Sampling frame: census and Geographic Information System databases;

- Sampling method: stratified random sample with gender ratio 1:1;

- Exclusion criteria: belonging to stakeholder level I to III;

- Sample size: n = 164 per site, n = 328 per phase

#### Level IV: phase 2 (post-vaccination)

##### Strategy

Community residents will be approached on both islands and information collected through EMIC interviews. Data will be collected i) to clarify the local features of cholera and shigellosis after vaccination, ii) to compare these features with pre-vaccination data, iii) to identify social and cultural determinants of actual OCV acceptance, and iv) to identify barriers to vaccination. The approximate duration of this phase is about ten weeks. Three teams each per site will be working simultaneously. This phase will be implemented after the end of the vaccination campaign.

##### Instrument

The EMIC interview will be exactly the same as in phase 1 except that a special section will enquire about the experience with the vaccination campaign and reasons against getting vaccinated among both unvaccinated and vaccinated respondents.

The interview teams will attend a second week-long workshop where they can refresh their skills and where the new section of the interview will be explained. A second pilot testing phase will take place to adapt the instrument with regard to the new section.

##### Sampling

- Sample: adults (≥ 18 years) from the target areas;

- Sampling frame: vaccination campaign data (vaccinated people), census database after exclusion of vaccinated people (unvaccinated people);

- Sampling method: equally stratified random sample among confirmed vaccinated (two doses of OCV) and unvaccinated (one to two doses) people with gender ratio 1:1;

- Exclusion criteria: belonging to stakeholder level I to III, already interviewed in phase 1, member of household already interviewed;

- Sample size: n = 164 per site, n = 328 per phase

### Data management and analysis

#### Level I to III: rapid assessment data

The rapid assessment interviews and focus group discussions will generate lengthy textual material which will be tape-recorded, followed by transcription and translation. This information will be organised and analysed using the qualitative analysis software of Ethnograph 6.0. This programme will allow the data to be multiply coded, segmented and searched according to important and emerging variables and themes.

#### Level IV: qualitative data

Information from focus group discussions will be tape-recorded, followed by transcription and translation. Narratives from EMIC interviews will be transcribed verbatim and translated. Typing will be done in a word processor software while MAXQDA 2007 will be used for managing the textual data and to facilitate analysis regarding findings from quantitative data.

#### Level IV: quantitative data

Information will be double-entered and cleaned in Epi Info 3.4.3 software. Descriptive statistics and bi- and multivariate analyses will be computed with the statistical analysis programme Stata 10.

Regarding the cholera vignette, the variables related to illness experience (PD), meaning (PC) and help-seeking behaviour (HS) will be coded with a value of two after a spontaneous response, a value of one after a probed response and a value of zero for no response at all to reflect the response style. A value of three will be assigned to the summary variables (i.e. most troubling, most important and most useful). For each category of such a variable, a total prominence will be computed, ranging from zero to five. Thematically similar individual categories will be grouped under specific headings (e.g. "physical symptoms" among PD variables) to enable the analysis of broader concepts of experience, meaning and behaviour. Grouped frequencies will be computed by adding the maximum value based on the response style of each respondent for every single category falling under each group. Calculation of the grouped prominence will follow the same procedure as with the individual variables.

For questions related to the shigellosis vignette, only frequencies of the categories mentioned in relation to illness experience, meaning and help-seeking behaviour will be computed.

Tabulations of the above-mentioned variables will be done after each phase followed by descriptive, bivariate and multivariate analyses to address the research questions.

To test for significant differences (p ≤ 0.05) between two groups, the t-test will be used for normal data and the Wilcoxon rank-sum test for nonparametric data. The Kruskal Wallis test will be used for comparing more than two groups of a nonparametric variable. The Chi^2 ^test and the Fisher's exact test will be applied for comparing two proportions. To compare paired data, i.e. variables between cholera and shigellosis vignettes, the McNemar's Chi^2 ^test, the paired t-test and the paired Wilcoxon test will be used.

Determinants of anticipated and actual OCV acceptance (outcome variables) will be identified in a twofold approach. First, bivariate tests (see above) will be done to see which variables are related with the outcome variables. Then, variables having a suggestive bivariate relationship (p ≤ 0.3) with the outcome variables will be retained as potential explanatory variables for computing stepwise logistic regression models.

Narrative accounts will be used to clarify and substantiate the relationships identified in the above analyses.

#### Data safety and storage

A password-secured data management system will be established where the responsible researchers on both islands can up- and download qualitative and quantitative data. Electronic data can only be accessed with permission from the co-investigator(s). Raw data (filled-in forms, narratives) will be kept under appropriate climatic and safety conditions in the Public Health Laboratory, Pemba. The data will be destroyed two years after the completion of the study (hard copies by incineration and soft copies by digital erasure).

### Translation of instruments and informed consent forms

The English versions of all the instruments and informed consent forms will go through cultural adaptation and translation into Kiswahili. Back-translation into English will ensure the validity of the translation and that no ethical alternation is introduced in the informed consent forms.

## Discussion

The study will clarify local perceptions of cholera and show how these and socioeconomic factors affect anticipated and actual acceptance of oral cholera vaccine in Zanzibar. The cross-sectional design and mixed-methods approach, attentive to the interests of various stakeholders, will help to explain critical practical features of cholera control. Cultural factors are widely regarded as important determinants of vaccine acceptance, but difficult to study. This study has formulated an approach that enables systematic consideration of their influence. Our findings and experience will guide the programme in Zanzibar. They will also show how research may enhance the effectiveness of interventions for cholera and other health problems in various settings where sociocultural and socioeconomic factors influencing population behaviour require careful study.

## Competing interests

The authors declare that they have no competing interests.

## Authors' contributions

CS participated in the conception and design of the study and drafted the manuscript. RH initiated the study, helped to design and coordinate it and to draft the manuscript. SMA and AMK helped in conception, design and coordination of the study and revised the manuscript. AP was involved in conception and design of the study and revised the manuscript. MGW contributed to the formulation and design of the study, and a critical review and revision of the manuscript. CLC initiated the study and participated in the design and revised the manuscript. All authors read and approved the final manuscript version.

## Pre-publication history

The pre-publication history for this paper can be accessed here:


